# Co-expression of 1α-hydroxylase and vitamin D receptor in human articular chondrocytes

**DOI:** 10.1186/s12891-017-1791-y

**Published:** 2017-11-06

**Authors:** Ann Kristin Hansen, Yngve Figenschau, Inigo Zubiaurre-Martinez

**Affiliations:** 1 Department of Orthopaedic Surgery, University Hospital of North Norway, Tromsø, Norway; 2 Bone and joint research group, Institute of Clinical Medicine, Faculty of Health Sciences, University of Tromsø, Tromsø, Norway; 3 Department of Laboratory Medicine, University Hospital of North Norway, Tromsø, Norway; 4 Endocrinology Research Group, Institute of Clinical Medicine, Faculty of Health Sciences, University of Tromsø, Tromsø, Norway; 5 Department of Medical Biology, Faculty of Health Sciences, University of Tromsø, Tromsø, Norway

**Keywords:** Vitamin D, 1α,25(OH)_2_D_3_, 25(OH)D_3_, VDR, CYP27B1, Chondrocyte, Cartilage, Osteoarthritis, Pellet culture, Static suspension culture

## Abstract

**Background:**

The aim was to investigate whether resident chondrocytes in human articular cartilage and in subculture express vitamin D receptor (VDR) and the enzyme that hydroxylates the prohormone 25(OH)D_3_ to the active hormone 1α,25(OH)_2_D_3_, namely 1α-hydroxylase (CYP27B1). Any putative effects of vitamin D on chondrocytes were also explored.

**Methods:**

Cartilage from human osteoarthritic knee joints, cultured chondrocytes and cells grown in 3D spheroids were examined for the expression of VDR and 1α-hydroxylase by PCR, Western blots and immunolabelling. Receptor engagement was judged by visualizing nuclear translocation. The effects of 25(OH)D_3_ and 1α,25(OH)_2_D_3_ on chondrocyte functions were assessed in proliferation-, chondrogenesis- and cartilage signature-gene expression assays. The capability of chondrocytes to hydroxylate 25(OH)D_3_ was determined by measuring the concentration of metabolites. Finally, a putative regulation of receptor and enzyme expression by 1α,25(OH)_2_D_3_ or interleukin (IL)-1β, was investigated by Western blot.

**Results:**

Gene expression was positive for VDR in freshly isolated cells from native cartilage, cells subcultured in monolayers and in spheroids, whereas protein expression, otherwise judged low, was apparent in monolayers. Nuclear translocation of VDR occurred upon 1α,25(OH)_2_D_3_ treatment. Transcripts for 1α-hydroxylase were detected in freshly isolated cells, cultured cells and spheroids. Western blots and immunolabelling detected 1α-hydroxylase protein in all materials, while staining of tissue appeared confined to cells at the superficial layer. A dose-dependent 1α,25(OH)_2_D_3_ production was measured when the enzyme substrate was supplied to cell cultures. Western blots revealed that the VDR, but not 1α-hydroxylase, was induced by IL-1β treatment in adherent cells. Proliferation in monolayers was enhanced by both 25(OH)D_3_ and 1α,25(OH)_2_D_3_, and both compounds had negative effects on chondrogenesis and cartilage-matrix genes.

**Conclusions:**

VDR expression in resident cartilage chondrocytes, generally considered differentiated cells, is elusive. A similar pattern applies for redifferentiated chondrocytes in spheroid cultures, whereas dedifferentiated cells, established in monolayers, stably express VDR. Both 25(OH)D_3_ and 1α,25(OH)_2_D_3_ are able to potentiate cell proliferation but have a negative impact in proteoglycan synthesis. Chondrocytes express 1α-hydroxylase and may contribute to the production of 1α,25(OH)_2_D_3_ into the joint environment. Effects of vitamin D could be unfavourable in the context of cartilage matrix synthesis.

**Electronic supplementary material:**

The online version of this article (10.1186/s12891-017-1791-y) contains supplementary material, which is available to authorized users.

## Background

Osteoarthritis (OA) is a debilitating degenerative disease of the joint affecting middle-aged and older people and the pathogenesis is considered multifactorial [1]. Over the last decades, mounting evidence suggests that OA develops as a cause of inflammation in addition to the mechanical aspects [2]. Vitamin D is a predecessor of the secosteroid hormone 1α,25(OH)_2_D, which plays a pivotal role for appropriately regulated calcium required for a normal bone turnover. Of importance, 1α,25(OH)_2_D has been suggested to dampen inflammation-driven diseases such as rheumatoid- and osteoarthritis [3]. Exogenous sources of vitamin D_3_ are mainly fatty fish and fortified food items, whereas the predominant source is the endogenous production from cholesterol in sun-exposed skin. After hydroxylation in the liver, the vitamin circulates as the prohormone 25(OH)D_3_ bound to D-binding protein (90%) and albumin (10%). Likewise, the active hormone 1α,25(OH)_2_D_3_ is transported by these proteins, but circulates in a concentration < 0.1% of that of the prohormone. Less than 1% of both metabolites are unbound by carrier proteins. The hydroxylation of 25(OH)D_3_ to 1α,25(OH)_2_D_3_ is facilitated by the enzyme 1α-hydroxylase, which is primarily located in the kidneys. However, there is compelling evidence of an extra-renal distribution of the enzyme, including local induction by the immune system [4]. The effect of vitamin D in the extra-renal compartments are considered auto- or paracrine, and represent an entirely new paradigm of vitamin D actions compared to the endocrine action of renal vitamin D [5]. The active hormone binds the vitamin D receptor (VDR), which is abundantly expressed in the intestine, kidneys, parathyroid and bone; while it is expressed in costal cartilage, its presence in articular cartilage is less settled [6].

Several studies have suggested a link between vitamin D status and OA; some have found that individuals deficient in vitamin D have an increased risk of progressive knee-osteoarthritis, and there are reports of a pain reducing effect of oral supplementation [7, 8]. Both 25(OH)D_3_ and 1α,25(OH)_2_D_3_ have been detected in synovial fluid [4, 9]. The presence of the receptor in human articular cartilage and chondrocytes has been previously investigated with divergent outcomes [10, 11], and the biological effect of vitamin D on cartilage pathophysiology remains uncertain. In rat chondrosarcoma cells, 1α,25(OH)_2_D_3_ dose-dependently induced MMP13, a matrix metalloprotease which degrades the extracelluar matrix [12]. 1α,25(OH)_2_D_3_ has also been associated with hypertrophy and mineralization of OA chondrocytes [13].

The aim of this study was to investigate whether the previously described expression patterns of VDR in human articular cartilage and subcultured cells could be confirmed by PCR, Western blots and immunolabelling. It was also questioned whether receptor activation occurred upon ligand binding, and whether this could be recorded by biological readouts such as promoted chondrogenesis, cell proliferation or cartilage signature gene expression. Furthermore, in view of the previously reported presence of 1α,25(OH)_2_D_3_ in synovial fluid, it was addressed whether the sole source was the circulation or if the hormone could also be attributed to a local production by chondrocytes expressing 1α-hydroxylase. Previous experiments are described using cartilage tissues and chondrocytes expanded in adherent monolayer cultures. Since chondrocytes are known to dedifferentiate upon monolayer expansion, thus undermining the extrapolation of findings to cartilage conditions, we included assays utilizing freshly isolated chondrocytes in a differentiated stage, and 3D culture assays where the chondrocyte redifferentiate to a chondrocyte-like phenotype [14].

## Methods

### Human material

Cartilage tissues were collected from macroscopically healthy looking areas of cartilage on the lateral femoral condyle of patients with osteoarthritis undergoing total knee replacement procedures (*N* = 13, age: 40–78). Patient records were searched to exclude any patients with rheumatic disease, while both secondary posttraumatic and primary osteoarthritis patients were included. The material was collected with the patient’s written consent and all samples were anonymized at time of collection. All methods were performed in accordance with the relevant guidelines and regulations, and the study was approved by the regional ethics committee (2015/1730/REK Nord).

### Chondrocytes isolation and culturing

#### Suspension cultures

Cartilage biopsies, in which bone was meticulously excluded, were minced into ~1mm^3^ pieces and enzymatically digested in Collagen type XI (Cat. no. C9407, Sigma Aldrich) for 4–6 h until 95% degraded. Cells were counted using a haemocytometer, and suspension cultures were established in 24-well ultra-low binding plates (Cat. no. 3473, Corning, VWR) at 4 × 10^5^ cells/well in DMEM (Cat. no. D5796, SigmaAldrich) supplemented with 62 mg/L ascorbic acid (Cat. no. 103033E, BDH Laboratories), 1% penicillin/streptomycin (P/S, Cat. no. P0781, Sigma-Aldrich) and 1% Fetal Bovine Serum (FBS, Cat. no. S0615, Biochrom). Suspension cultures were allowed to equilibrate for 24 h in at 37 °C and 5% O_2_ before being treated with IL-1β (Cat. no. 200-01B, Peprotech) at 10 ng/mL, 1α,25(OH)_2_D_2_ (Cat. no. 11174, Cayman Chemical) or 1α,25(OH)_2_D_3_ (Cat. no. 71820, Cayman Chemical) at 10^−8^ M for 24 h.

#### Monolayer cultures

Immediately after tissue digestion, chondrocytes were seeded in polystyrene T25 flasks and serially expanded in monolayer cultures in DMEM supplemented with ascorbic acid, P/S and 10–20% FBS until confluence in T175 cultures flasks. Monolayer cells were used for experimentation at passages 2–5. Pieces of bone removed from cartilage biopsies were minced and explant cultured in DMEM supplemented with 20% FBS for 2 weeks to obtain the osteoblasts used as controls in Western blots.

#### 3D cultures

Scaffold-free multicellular spheroid cultures of chondrocytes were established from chondrocytes expanded in the presence of vehicle, 25(OH)D_3_ or 1α,25(OH)_2_D_3_ at 10^−7^ M until confluency. The cells were detached from the culture flasks using an enzyme-free dissociation solution (Cat. no. S-014-B, Merck Millipore) and transient trypsinization (Trypsin-EDTA, T4049, Sigma-Aldrich) before plating in round bottom ultra-low attachment 96-well plates (Cat. no. 7007, Costar) at a density of 5 × 10^4^ in 150 μL DMEM supplemented with 10 ng/mL TGF-β3 (Cat. no. 100-21C, Peprotech), 10 ng/mL BMP-2, 1:1000 Insulin-Transferrin-Selenium (ITS, Cat. no. 354351, BD Biosciences). The augmentation with vehicle, 25(OH)D_3_ or 1α,25(OH)_2_D_3_ at 10^−7^ M was continued through 3D culturing for 2 weeks for spheroids used to evaluate chondrogenesis. Spheroids used for immunolabelling of VDR and 1α-hydroxylase were propagated for 2 days or 3 weeks in chondrogenic medium alone.

### Histology and immunohictochemistry

Cartilage biopsies, two-day spheroids and three-week spheroids were fixed in 4% formalin overnight. Spheroids were embedded in 1% agarose before, along with the cartilage biopsies, they were cast and cut into 4 μm sections. Slides were rehydrated and antigen retrieval was achieved by placing the slides in a 60 °C citrate buffer for 60 min. The SuperPicture kit (Cat. no. 879673, Novex, LifeTechnologies) was used, along with specific antibodies targeting 1α-hydroxylase (Cat. no. ABIN2118284, Antibodies-online.com) and VDR (Cat. no. Sc-13,133, Santa Cruz) [15]. As previously described [16], slides from two-day and three-week controls and vitamin D-treated spheroids were prepared and stained with Alcian blue to detect glycosaminoglycan production. Pictures were developed using the Zeiss Axiophot photomicroscope (Carl Zeiss, Oberkochen, Germany), and the images were evaluated by three investigators using the Bern score to assess chondrogenicity [17].

### Immunofluorescence

Chondrocytes were cultured until confluency seeded on 2-well chamber slides (Cat. no. 177429; NUNC Lab Tek), and incubated for 24 h before being treated with vehicle or 1α,25(OH)_2_D_3_ at 10^−8^ M for 2 h, and subsequently subjected to fixation. Concomitant fixation-permeabilization of cells was done using 100% ice-cold methanol for 8 min, followed by serial washings with PBS for the rehydration of cells. For protein detection, fixed cells were incubated with primary antibodies against 1α-hydroxylase and VDR. Alexa 546-conjugated secondary antibodies targeting rabbit (Cat. no. A11010, Life Technologies) or mouse IgG (Cat. no. A11003, Life Technologies), respectively, were used to visualize 1α-hydroxylase and VDR in an appropriate microscope.

#### Western blot

Protein was extracted from freshly isolated chondrocytes, suspension cultures and monolayer- expanded cultures using the NP-40 buffer (150 nM NaCl, 50 mM Tris-HCl, 1% Igepal), supplemented with protease inhibitor (Complete, EDTA-free, Cat. no. 11873580001, Roche). Protein concentration was measured using a colorimetric assay (Cat. no. 500-0116, BioRad), and separated along with BLUeye Prestained Protein Ladder (Cat. no. PM007-0500, Sigma-Aldrich) and MagicMark™ XP Western Protein Standard Ladder (Cat. no. LC5602, Novex, Life Technologies) using TruPage gels (Cat. no. PCG2003, Sigma-Aldrich). The protein input was 40 μg/lane for cartilage samples, 45 μg/lane for suspension cultures and 20 μg/lane for cultured cell samples. Proteins were transferred to PVDF membranes, incubated for 1 h in a dry milk buffer and incubated overnight at 4 °C with 1α-hydroxylase (1:200 in BSA buffer) or VDR antibody (1:100 in dry milk buffer). Next, the membranes were incubated with secondary α-rabbit antibody (Cat. no. sc-2004, SantaCruz) and α-mouse antibody (Cat. no. sc-2005, SantaCruz) for 1 h at room temperature, and lastly, a chemiluminescence detection solution (Cat. no. 170-5040, BioRad) was applied before images were procured using an ImageQuant LAS 4000 CCD camera. Beta-actin antibody (Cat. no. SAB5500001, Sigma-Aldrich) was used as a loading control, relative density was assessed using Image Studio Lite 5.2 and a Dunnett’s test used to compare the treated groups to the control group.

### PCR

Suspension cultured cells and three-weeks spheroid cultures were harvested, and RNA was extracted using the RNeasy Micro Kit (Cat. no. 74004, Qiagen). Lysates were dissolved in TissueLyser (Qiagen), homogenized using QiaShredder columns (Cat. no. 79654, Qiagen), and cleaned and eluted according to the manufacturer’s instructions. RNA from monolayer expanded chondrocytes was extracted using the PerfectPure Cultured Cell Kit (Cat. no. 2900319, 5prime) according to the manufacturer’s protocol. RNA concentration was measured by Nano-Drop 2000 and 35 ng of each sample was reverse-transcribed to cDNA using qScript (Cat. no. 95047, Quanta Biosciences, VWR). The PCR reaction included JumpStart REDTaq ReadyMix, cDNA and specific primers targeting 1α-hydroxylase (CYP27B1) and VDR (Table 1), and was amplified for 35 cycles using an MJ Research thermal cycler. Products were separated in FlashGels (Cat. no. 57023, Lonza, Fisher Scientific), and images were obtained using an ImageQuant LAS4000 camera system.

**Table 1:** PCR Primers

VDR F	5′-ACC AAG CTC ACA GTT CCT CG-3′
VDR R	5′-CGG CAG GGA GAT CAT GAC TC-3′
CYP27B1 F	5′-ACC ATG GTC TCT CTG CTT GC-3′
CYP27B1 R	5′-GCC CAA AGA TGT CTC TGC CT-3′
APRT F	5′-CCCGAGGCTTCCTCTTTG GC-3′
APRT R	5′-CTCCCTGCCCTTAAGCGAGG-3′

### qPCR

RNA was extracted from monolayer cultures propagated in 6-well plates and treated with vehicle, 25(OH)D_3_ or 1α,25(OH)_2_D_3_ at 10^−6^ M and 10^−7^ M for 72 h. Extracts were used in a qPCR assay where each reaction contained 5 μL Precision Fast ROX MasterMix (Primer Design), 0.5 μL hydrolysis probe (Invitrogen), 2.5 μL H_2_O and 2 μL cDNA. Hydrolysis probes (Life Technologies by Fisher Scientific) include: collagen type I alpha 1 chain (*COL1A1*, Assay ID: Hs00164004_m1), collagen type II alpha 1 chain (*COL2A1*, Hs00264051_m1), aggrecan (*ACAN*, Hs00153936_m1), versican (*VCAN*, Hs00171642_m1), SRY-box 9 (*SOX9*, Hs00165814_m1), cytochrome P450 family 24 subfamily A member 1 (CYP24A1, Hs00167999_m1), vitamin D receptor (VDR, Hs01045843_m1) and ribosomal protein L13a (*RPL13A*, Hs04194366_g1, reference gene). Reactions were run in BrightWhite 96-well plates (PrimerDesign) using a StepOnePlus™ Real-Time PCR System. The dCq was calculated by subtracting the reference gene from the gene of interest so that a higher dCq value represents an upregulation and vice versa. A Dunnett’s test was used to compare treated samples to the control samples.

### 25(OH)D_3_ hydroxylation assay

To detect the hydroxylation of 25(OH)D_3_ to 1α,25(OH)_2_D_3_, parallel 6-well plates with and without confluent chondrocytes were challenged with 25(OH)D_3_ at 0, 50, 250 and 500 nM for 24 h. Supernatants were collected, and 1α,25(OH)_2_D_3_ was measured using an automated immunometric assay (Diasorin) at the Hormone Laboratory, Oslo University Hospital, Norway. Conversion attributed to 1α-hydroxylase activity was calculated as the difference between 1α,25(OH)_2_D_3_ in wells with and without cells in order to cancel out any spontaneous conversion. A linear model was used to evaluate the correlation between 25(OH)D_3_ and 1α,25(OH)_2_D_3_.

### Proliferation assay

Chondrocytes from two donors were plated in E-plate VIEW 16 (Cat. no. 06324746001, ACEA Biosciences) at 3000 cells per well in 100 μL DMEM, supplemented with 10% FBS and vehicle, 25(OH)D_3_ or1α,25(OH)_2_D_3_ at 10^-6^ M for 80 h and real-time proliferation was monitored using the xCELLigence RTCA system (Roche Diagnostics). The cell-index was normalized to 25 h (1 h after treatment) and log 2 converted to more accurately display growth rate [18].

#### Immunoelectron microscopy

Chondrocytes cultures from two different donors established in 10 cm Ø dishes were fixed in 8% formaldehyde in a 200 mM HEPES buffer, pH 7.5, for 24 h, collected by gently scraping of cells and centrifuged in an Eppendorf microfuge. After infusion for 30 min with 2.3 mol/L sucrose containing 20% polyvinylpyrrolidone, pellets were mounted on a specimen holder and frozen in liquid nitrogen. Cryosections were prepared, and immunolabeling was performed as described elsewhere [19]. Antibodies against VDR and 1α-hydroxylase (diluted 1:25) were detected by protein A-gold complexes, and the dried sections were then examined in a JEOL JEM 1010 transmission electron microscope (JEOL, Tokyo, Japan) operating at 80 kV. Moreover, negative controls were routinely included in parallel by the omission of primary antibodies.

### Statistics

Data analysis and figures were acquired using RStudio [20], with tidy, plyr, dplyr, broom, data.table, purrr, ggplot2 and ggthemes packages [21–28]. Differences were considered significant at *p* < 0.05.

## Results

### Chondrocyte dedifferentiation and redifferentiation

In this study, we have investigated the expression of vitamin D receptor and 1a-hydroxylase on human articular chondrocytes established in different experimental conditions comprising native tissue, suspension cultures, adherent cells/monolayer cultures and 3D spheroids cultures. This approach is relevant if we take into consideration that chondrocytes change their phenotype during cell expansion in vitro (dedifferentiation) and that they are able to gain (at least in part) their phenotypic traits in 3D cultures (redifferentiation) [14]. A qPCR assay was performed, comparing gene expression of cartilage signature genes between suspension cultures and monolayer cultures, and the results indicate that the suspension cultures were indeed in a differentiated stage compared to the dedifferentiated monolayers (Additional file 1: Figure S1).

### Expression of 1α-hydroxylase in cartilage tissue and cells

**Fig. 1:**
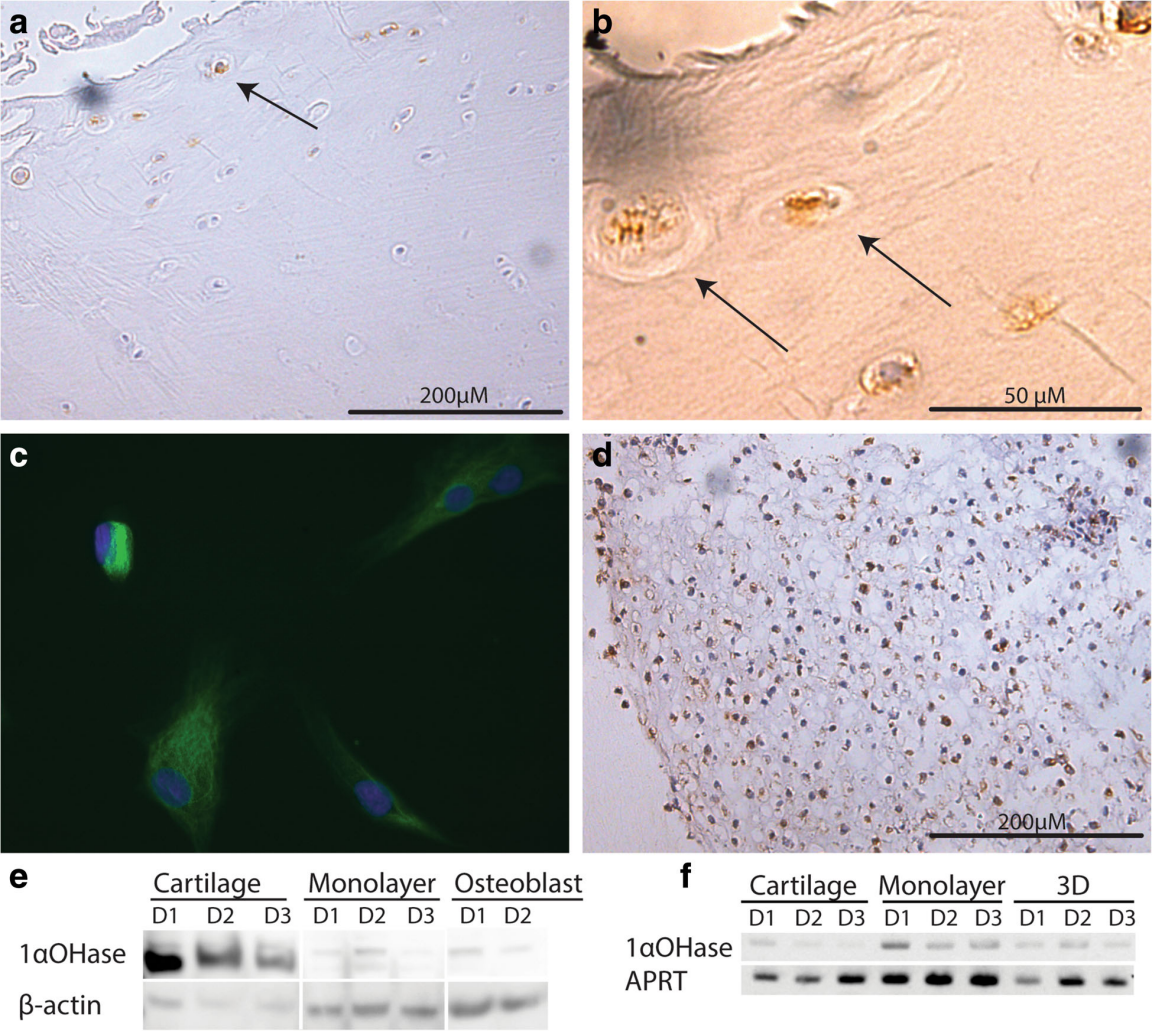
Cartilage at 100 × (**a**) and 400 × (**b**) magnification, arrows indicate chondrocytes labelled with 1α-hydroxylase antibody and corresponding green labelling of cultured chondrocytes (**c**). In the two-week spheroids (**d**, 100×) brown staining of chondrocytes indicate the presence of 1α-hydroxylase. Western blot (**e**) of cartilage, monolayer chondrocytes and osteoblasts labelled with 1α-hydroxylase antibody (58 kD) and β-actin (45 kD, loading control). PCR (**f**) of cartilage, monolayer chondrocyte and spheroids (3D) using primers targeting 1α-hydroxylase (521 bp) and APRT (300 bp, quality control). Images represent the outcome of investigation of cells and tissues from three different donors

By using conventional PCR, 1α-hydroxylase mRNA was detected in freshly isolated cells from native cartilage, chondrocytes in monolayers and in 3D spheroids cultivated for 3 weeks in chondrogenic conditions (Fig. 1f). By immunolabelling of native cartilage, 1α-hydroxylase appears confined to chondrocytes residing in the superficial layer (in Fig. 1a and b). Chondrocytes in monolayers and in spheroids were also positively stained (Fig. 1c and d). In Western blots, an expected 58 kD band in protein extracts from native cartilage, suspension and monolayer cells as well as in a positive control, i.e. osteoblast culture, was confirmed (Fig. 1e). In subcellular distribution experiments, utilizing immunoelectron microscopy, 1α-hydroxylase was found in the cytoplasm and in electron-dense mitochondria-like structures (Fig. 3b).

### Expression of VDR in cartilage tissue and cells

**Fig. 2:**
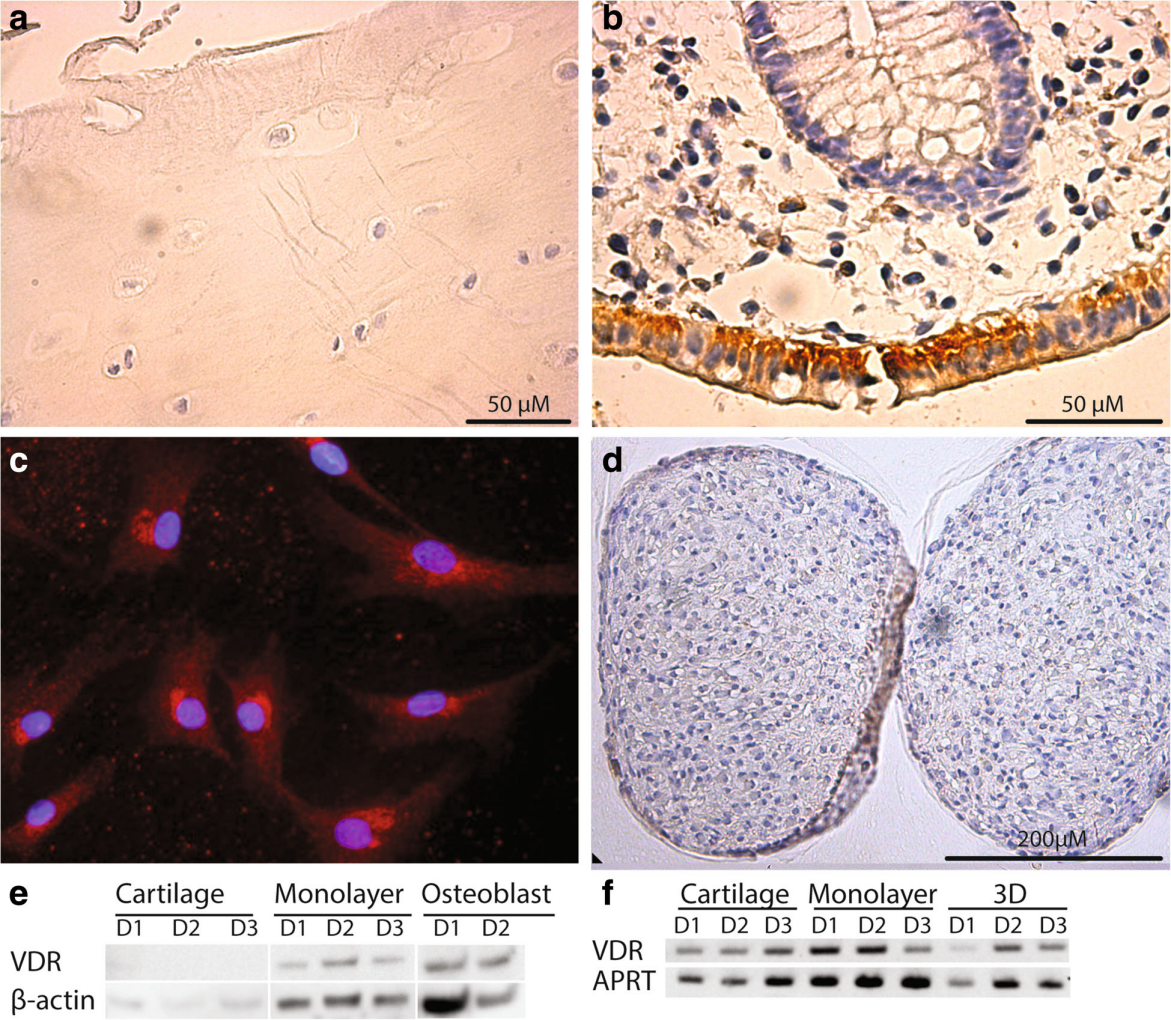
Cartilage (**a**, 400×), normal colon (**b**, 400×), cultured chondrocytes (**c**) and two-day spheroids (**d**, 100×) labelled with a VDR antibody. In normal colon the brown staining of the cytoplasm represent VDR labelling and in cultured chondrocytes the VDR is correspondingly visualised by red staining, while in cartilage and two-week spheroids there is no labelling of VDR. Western blot (**e**) of cartilage, monolayer chondrocytes and osteoblasts labelled with VDR antibody (53 kD) and β-actin (45 kD). PCR (**f**) of cartilage, monolayer chondrocytes and spheroids (3D) using primers targeting VDR (336 bp) and APRT (300 bp). Images represent the outcome of investigation of cells and tissues from three different donors

**Fig. 3:**
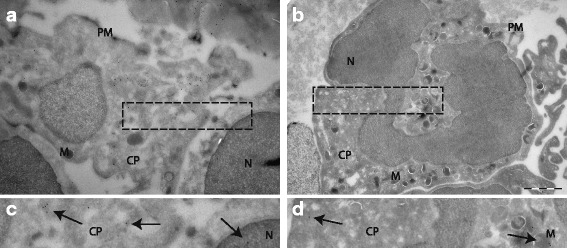
Subcellular distribution of VDR (**a**) and 1α-hydroxylase (**b**) in chondrocytes assessed by immunoelectron microscopy. Panels **c** and **d** are magnifications of **a** and **b** respectively. Gold particles are marked with arrowheads. N = nucleus, M = mitochondria, PM = plasma membrane, CP = cytoplasm

Results show that mRNA transcripts encoding VDR was detected in all samples, i.e. in freshly isolated chondrocytes, monolayer cultured cells and three-weeks spheroids (Fig. 2f). As judged by immunolabelling, no staining of the VDR protein was identified in cartilage (Fig. 2a) in contrast to the staining observed in intestinal mucosa used as a positive control (Fig. 2b). In monolayer expanded cells, staining was apparent in the cytoplasm and the nucleus (Fig. 2c). In 3D spheroid cultures, immunostaining was negative already 2 days after 3D culture initiation (Fig. 2d). Western blotting confirmed these findings by the occurrence of an expected 53 kD band in extracts from monolayer cells and the positive control, primary osteoblasts culture (Fig. 2e). Of note, the nearly invisible band corresponding to the loading control beta-actin in cartilage samples underscores the low levels of cell-associated proteins compared to extracellular matrix (ECM) proteins in such samples. At the subcellular level, VDR was found primarily in cytoplasmic regions and sporadically in the nucleus (Fig. 3a).

### Receptor and enzyme are functional

**Fig. 4:**
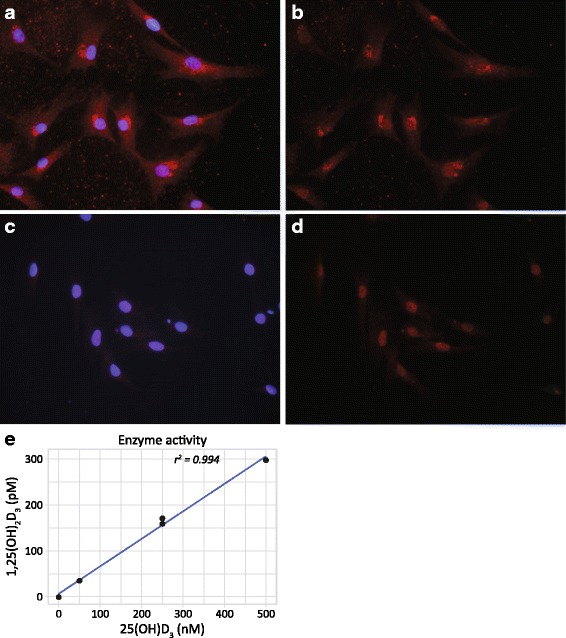
Cultured chondrocytes labelled with VDR antibody (**b** and **d**) and merged image of DAPI and VDR labelling (**a** and **c**). Cells in **a** and **b** are untreated, while **c** and **d** are treated with 1α,25(OH)_2_D_3_ for 2 h. Cultured chondrocytes treated with 50, 250 or 500 nM 25(OH)D_3_ for 24 h compared to level of 1α,25(OH)_2_D_3_ measured in the supernatant (**e**), results represent the experiment on cells from one donor

VDR engagement was investigated by immunolabelling of nuclear translocation of the receptor upon ligand binding. In Fig. 4c and d, the predominantnuclear staining of VDR after 2 h of treatment with 1α,25(OH)_2_D_3_ can be observed, compared to the mixed cytoplasmic-nuclear staining of VDR from untreated cells (Fig. 4a and b).

Evidence for 1α-hydroxylase activity was arranged by supplementing cultured cells with its substrate 25(OH)D_3_ followed by the assessment of 1α,25(OH)_2_D_3_ concentration after 24 h. It was revealed that 1α,25(OH)_2_D_3_ was dose-dependently produced in cell supernatants (Fig. 4e).

### Induced expression of VDR, but not 1α-hydroxylase by IL-1β

**Fig. 5:**
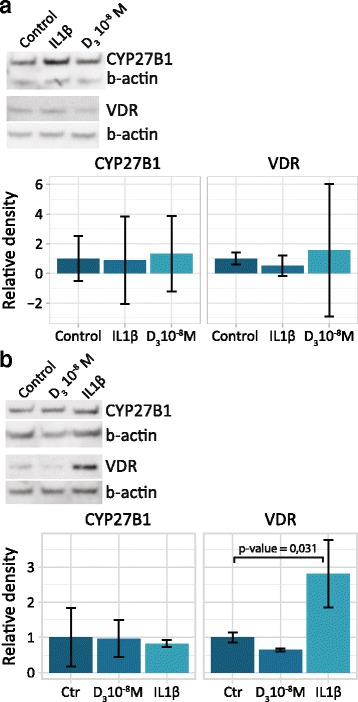
Western blots targeting VDR and 1α-hydroxylase in chondrocyte suspension cultures (**a**) and monolayer cultures (**b**) exposed to IL-1β (10 ng/mL) or 1α,25(OH)_2_D_3_ (10^−8^ M) for 24 h; controls are untreated cells. Graphs show the corresponding protein expression by densitometry in samples from three different donors. Error bars represent 95% CI

Aiming at exploring potential receptor and/or enzyme regulation during inflammatory conditions, VDR and 1α-hydroxylase expression was judged by Western blotting in freshly isolated cells and in monolayers after treatment with the pro-inflammatory cytokine IL-1β or the active hormone 1α,25(OH)_2_D_3_. Bands occurring after blotting from three randomly chosen donors were subjected to densitometric assessment. In suspension culture preparations, an increased expression of 1α-hydroxylase (CYP27B1) was detected in two out of three samples, but the lack of regulation in one donor made the total effect not significant. In contrast, the expression of VDR was uniform across treated and untreated samples (Fig. 5a). In monolayer samples, the VDR expression was amplified almost three-fold after IL-1β stimulation, an alteration that was judged as statistically significant (Fig. 5b), while the expression of 1α-hydroxylase was unaltered by IL-1β or 1α,25(OH)_2_D_3_.

### Both 25(OH)D_3_ and 1α,25(OH)_2_D_3_ influence chondrogenesis and expression of cartilage signature genes

**Fig. 6:**
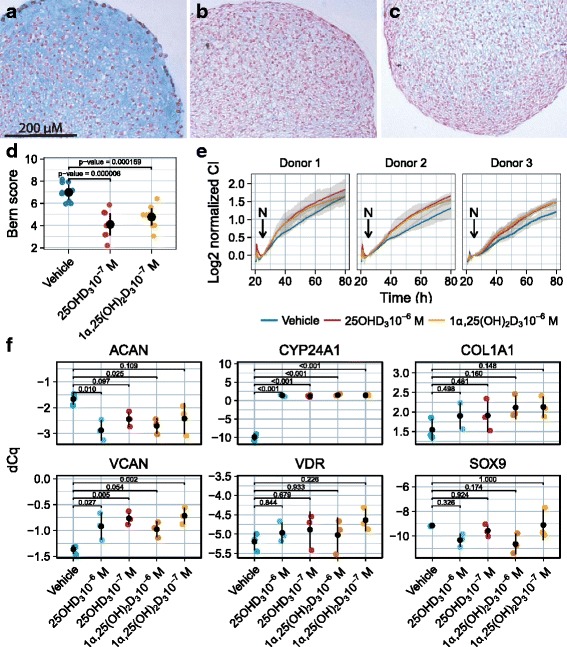
Alcian blue staining of spheroids maintained in control medium (**a**) 25(OH)D_3_ (**b**) or 1α,25(OH)_2_D_3_ at 10^−7^ M (**c**) for 2 weeks. Treated and untreated spheroids from three different donors histologically evaluated by Bern Score (**d**). Proliferation (**e**) of monolayer chondrocytes under continuous stimulation with 25(OH)D_3_, 1α,25(OH)_2_D_3_ or vehicle for 80 h in three donors. Ribbons represent 95% confidence interval and N indicates the time of normalization. Cartilage signature gene expression in monolayer cultures from three donors (**f**). Error bars represent one standard deviation, while horizontal bars and values represent *p*-values resulting from the comparison of means of the treated samples to the mean of the vehicle samples

Last, the potential effects of 25(OH)D_3_ and 1α,25(OH)_2_D_3_ on major chondrocyte functions was investigated. Since the expression of the VDR receptor was more evident in monolayer cells, and scarcely detectable in freshly isolated or 3D conditions, we resorted to chondrocytes in monolayers to study vitamin D effects. For chondrogenesis, cells established in monolayers were incubated in the presence or absence of 25(OH)D_3_ or 1α,25(OH)_2_D_3_ for 1 week prior to 3D spheroids formation and incubation for 2 weeks in a chondrogenic environment supplemented with 25(OH)D_3_, 1α,25(OH)_2_D_3_ or vehicle. The resulting 3D structures were stained with the glycosaminoglycan marker Alcian blue (Fig. 6a-c). A semi-quantitative visual scoring of Alcian blue-stained spheroids from three different donors (Bern score) indicated a detrimental effect of both 25(OH)D_3_ and 1α,25(OH)_2_D_3_ on proteoglycan synthesis and chondrogenesis (Fig. 6d).

Effects of 25(OH)D_3_ or 1α,25(OH)_2_D_3_ on cartilage-signature gene expression were studied by qPCR (Fig. 6f). Regulation of CYP24A1 expression was used as an internal positive control for 1α,25(OH)_2_D_3_ action. Cultures treated with 25(OH)D_3_ or 1α,25(OH)_2_D_3_ both exhibit a significant increase in expression of CYP24A1 compared to the untreated cultures, while the VDR transcript remained unaffected. Both 25(OH)D_3_ and 1α,25(OH)_2_D_3_ significantly increased the expression of VCAN, whereas the expression of ACAN (the cartilage proteoglycan) was significantly reduced compared to the control. The expression of Collagen type I (COL1A1) and SOX9 were not affected by 25(OH)D_3_ nor 1α,25(OH)_2_D_3_ treatment. Expression of Collagen type II remained undetectable in both treated and untreated samples.

On the other hand, we found that both 25(OH)D_3_ and 1α,25(OH)_2_D_3_ had a positive impact on cell proliferation, both inducing a slight increase in the growth rate compared to vehicle (Fig. 6e). The three donors included in the study exhibited different growth rates, obscuring any significant differences.

## Discussion

Osteoarthritis (OA) is a disease of the entire joint affecting cartilage, subchondral bone, the synovial membrane and ligaments. Clinically, the disease is characterized by joint space narrowing, osteophyte formation and sclerosis. At a microscopic level, OA is characterized by a loss of extracellular matrix, chondrocyte hypertrophy, cell proliferation and calcification [29]. The role of Vitamin D in the OA context remains controversial. Synoviocytes secrete inflammatory mediators into the synovial fluid, and it appears that elevated levels of 1α,25(OH)_2_D_3_ may increase the OPG/RANKL ratio, along with a reduced IL-6 production that together contributes to a dampened inflammation [30]. In contrast, it was reported that in human articular chondrocytes, 1α,25(OH)_2_D_3_ induced MMPs and calcification that are usually considered detrimental events [11, 13]. The picture becomes more entangled after considering the studies that have investigated VDR expression in human cartilage. An early study from the 80’s claimed that VDR is absent in resident cartilage cells, but acquires VDR during ex-vivo cultivation, i.e. cells considered as dedifferentiated [10]. A more recent report argues that VDR is inconsistently expressed in healthy human cartilage (donor dependent), but enhanced in OA cartilage [11]. Because of the existing dubiety, we pursued studying VDR expression in cartilage tissue and cells by different means. Preceded by the measurement of 25(OH)D_3_ in synovial fluid from patients with rheumatoid arthritis (mean 11,0 nM, results not shown), it was importunate to question whether the congeneric 1α,25(OH)_2_D_3_ hormone could be produced locally in the joint by chondrocytes. This would require the recognition that chondrocytes express 1α-hydroxylase.

In line with previous publications [10, 31], VDR transcripts were detected in cartilage tissue and monolayers, while in our hands detecting VDR protein in native cartilage by immunohistochemistry and Western blot was less evident. The expected VDR band is hardly recognizable in Fig. 2e (cartilage D1, the D2 and D3 are judged negative). Of note, the β-actin protein band from cartilage samples was also weak even though equal amount of proteins were loaded in wells. This may reflect a very low concentration of cell associated proteins compared to ECM proteins in samples prepared by short enzymatic digestion. In Fig. 5a, corresponding to the Western blot of suspension cells phenotypically similar to native cells and devoid of matrix proteins, it is more evident that the VDR protein is present in the samples. No previous publication could be recovered for a comparison on the Western blot subject, and our judgment is that VDR is expressed by resident cells in cartilage, although at very low levels.

Through immunolabelling it has previously been demonstrated that chondrocytes express the VDR protein in OA cartilage and monolayer cells [11]. The latter was confirmed here (Fig. 2c), though no signal was recorded by histological immunolabelling (Fig. 2a). It has been claimed that many VDR antibodies not only bind VDR, but also possess non-specific interactions with other unidentified proteins, determined by both immunoblotting and histochemistry [15]. These authors recommended using the antibody applied in the present study for both purposes. The questioned utility of different antibodies could explain the current divergent findings. All in all, despite the failure to detect the VDR protein in cartilage tissue by immunohistochemistry, the results from protein and transcript detection in suspension cell cultures represent an evidence that differentiated chondrocytes express VDR, which is also in agreement with both the previous report and reports on enhanced expression in OA cartilage and rheumatoid lesions [31]. In line with what we observed in tissue, a negative immunolabelling of redifferentiated cells, i.e. those in spheroids was recorded. Although VDR transcripts were detected, no or an insufficient amount of protein in spheroids enabled VDR detection.

After entering the cell, 1α,25(OH)_2_D_3_ binds the VDR, and the VDR-ligand complex translocates to the nucleus where it triggers a tissue-specific change in gene-transcription, resulting in altered growth, differentiation or functional activity [32]. In adherent chondrocytes, receptor translocation is evident in Fig. 4 a-d where the immunostaining shifts from a mixed nuclear/cytoplasmic stain in untreated chondrocytes, to a predominant nuclear staining after the addition of 1α,25(OH)_2_D_3_. This provides evidence of the internalization of 1α,25(OH)_2_D_3_ and subsequent receptor engagement.

An objective in this study was to investigate a putative alteration of receptor or enzyme expression during an inflammatory condition arranged by treating cells with cytokine or hormone. Cells in both suspension and monolayers were subjected to either IL-1β or 1α,25(OH)_2_D_3_ treatment, and relative amounts of VDR protein were recorded by Western blot. In monolayer samples, the VDR was significantly upregulated upon treatment with IL-1β (Fig. 5b), while this effect could not be detected in a suspension culture condition (Fig. 5a). The upregulation detected in monolayer cultures is in agreement with previous publications reporting upregulated VDR expression during various inflammatory conditions and could be associated to the elevated receptor expression reported in OA cartilage samples [31].

In osteoclasts, there has been observed an upregulation of VDR transcripts upon 1α,25(OH)_2_D_3_ stimulation at 10^−7^ M, but not at a 10^−8^ M level [33]. In the present study, 10^−8^ M was used to resemble the amounts found in the synovial fluid, yet it appeared insufficient to affect VDR expression at the protein level (Fig. 5). This indicates that a supplementary local hormone production is required [34], or that osteoblasts and chondrocytes are unrelated cells on this subject.

It is claimed that in general 1α,25(OH)_2_D_3_ has an anabolic effect on tissues [35]. In studies of proliferation, the mutual potency of 25(OH)D_3_ and 1α,25(OH)_2_D_3_ was proven by the changes observed with each of the compounds. A similar pattern was seen in three donors, indicating enhanced proliferation after 25(OH)D_3_ or 1α,25(OH)_2_D_3_ treatment (Fig. 5e). Chondrocyte proliferation is frequently observed in OA cartilage [29], possibly as an attempt of cells to repair the damaged cartilage or to compensate the catabolic processes established in the joint.

Moreover, from the spheroid model that investigates chondrogenic potential, a corresponding effect was observed resulting in a significant loss of matrix production (Fig. 6 a-d) during treatment with 25(OH)D_3_ or 1α,25(OH)_2_D_3_, but only after applying the assay to chondrocytes that also were expanded in the presence of 25(OH)D_3_ or 1α,25(OH)_2_D_3_. Spheroids prepared from chondrocytes propagated in standard growth medium were indifferent to the presence of 1α,25(OH)_2_D_3_ during 3D culture (data not shown), underpinning that chondrocytes in 3D cultures rapidly repress VDR expression.

The gene expression profile of cultured cells exposed to 25(OH)D_3_ and 1α,25(OH)_2_D_3_ indicated an unfavourable effect on proteoglycan transcripts ACAN and VCAN, while the expression of VDR, COL1A1 and SOX9 was unchanged. This outcome is in accordance with the lower expression of proteoglycans observed by Alcian blue staining of treated spheroids. Interestingly, the CYP24A1, that was included as a positive control of 1α,25(OH)_2_D_3_ effects, showed increased expression also upon 25(OH)D_3_ treatment (Fig. 6 f), supporting the notion that chondrocytes endogenously express 1α-hydroxylase.

Striking and novel findings in this study were the expression of transcripts for the enzyme 1α-hydroxylase and the presence of the encoded protein in human osteoarthritic articular cartilage, in suspension cells, in monolayers and 3D spheroid cultures (Fig. 1). The expression in all these conditions could indicate a constitutive expression pattern. No evidence for 1α-hydroxylase regulation by IL-1β or 1α,25(OH)_2_D_3_ in differentiated suspension cells or dedifferentiated monolayer cells was recorded, which is in line with previous studies on osteoblasts [33]. Previously, the enzyme was detected in rat growth plate chondrocytes [36], but to the best of our knowledge there are no reports on its presence in human articular cartilage or chondrocytes. Expression of 1α-hydroxylase in cartilage from healthy donors remains to be determined.

The level of 25(OH)D_3_ and 1α,25(OH)_2_D_3_ in the synovial fluid of inflamed joints in patients with rheumatoid arthritis has been measured to 20 nM and 25 pM, respectively [4]. After oral administration of vitamin D, the level of 1α,25(OH)_2_D_3_ in synovial fluid has been measured to up to 100 nM [37]. Based on these measurements, and to meet the sensitivity of the assay, chondrocytes were challenged with 50, 250 and 500 nM 25(OH)D_3,_ resulting in a conversion to 50, 150 and 300 pM 1α,25(OH)_2_D_3_, respectively (Fig. 4e). Since cell-free controls were subtracted to account for spontaneous 1α-hydroxylation, the results imply that the chondrocyte exhibits 1α-hydroxylase activity that may contribute to the pool of 1α,25(OH)_2_D_3_ in synovial fluid, an action that has previously been attributed solely to macrophages in synovial fluid [4, 38].

This study has provided strong evidence for 1α-hydroxylase being expressed in human articular chondrocytes, at least in OA-derived chondrocytes, whereas evidence for a VDR expression is weaker, except in culture-expanded cells. The activity of the 1α-hydroxylase was supported by the conversion of 25(OH)D_3_ to 1α,25(OH)_2_D_3_ (Fig. 4e) and indirectly by the assays presented in Fig. 6 showing comparable outcomes from treatment with 25(OH)D_3_ and 1α,25(OH)_2_D_3_. Hence, the co-expression of these proteins enables auto- and paracrine cell activity, exemplified here by impaired matrix production, augmented cell proliferation and shifted ACAN/VCAN expression after the application of either 1α,25(OH)_2_D_3_ or 25(OH)D_3_ (Fig. 6). These changes in chondrocyte activity are indeed associated with OA progression [39], however since the functional experiments were conducted on dedifferentiated cells, some caution is advised in extrapolating these results to in vivo conditions. On the other hand, as a result of the increased expression of VDR during inflammatory conditions reported by us and others [31], some of the actions described in Fig. 6 may be part of the picture during OA and RA pathology.

The extent and types of effects 1α,25(OH)_2_D_3_ have on resident cells in healthy cartilage remains to be uncovered, yet a probable paracrine action affecting neighbouring cells and tissues is apparent. However, the numerous VDR responsive elements in DNA and VDR’s capability to additionally engage several intracellular signalling systems, such as protein kinase C and phosphatidyl-inositol-3′ kinase reviewed in [40], vouch for a plethora of biological readouts beyond the scope of this study to be investigated. Thus, this study does not rule out any anti-inflammatory effects of vitamin D on the joint as a whole.

## Conclusion

To conclude, we report here a novel finding on the vitamin D converting enzyme, 1α-hydroxylase, in human articular chondrocytes along with compelling evidence of the enzyme facilitating the conversion of 25(OH)D_3_ to the active hormone, 1α,25(OH)_2_D_3_. The expression of the vitamin D receptor is more elusive and reproducible experiments are limited to monolayer conditions where the expression of the VDR is more evident. The overall effect of both 25(OH)D_3_ and 1α,25(OH)_2_D_3_ include diminished matrix production, enhanced proliferation and inversed expression of ACAN and VCAN, all pointing to an unfavourable effect of vitamin D on matrix synthesis.

## Additional file

Additional file 1: Figure S1. qPCR. Relative expression of cartilage signature genes in donor matched suspension- and monolayer culture samples from three donors. Suspension cultures samples were challenged with vehicle, 1α,25(OH)_2_D_2_ or 1α,25(OH)_2_D_3_ at 10^-8^ M for 24 hours. The qPCR reaction was setup as described in material and methods. Collagen type IX alpha 1 chain (COL9A1, Hs00932129_m1) and collagen typeX alpha 1 chain (COL10A1, Hs00166657_m1) hydrolysis probes (Life Technologies by Fisher Scientific) were included in addtion to probes listed previously. The dCq was calculated by subtracting the reference gene from the gene of interest so that a higher dCq value represents an upregulation and vice versa. Delta Cq values were mean-centred and autoscaled, and normalized to a suspension culture control sample to obtain ddCq [1]. A Dunnett’s test was used to compare the treated suspension cultures samples and the untreated monolayer samples to the suspension culture control sample. Error bars represent 95 % confidence intervals. References 1. Willems E, Leyns L, Vandesompele J. Standardization of real-time PCR gene expression data from independent biological replicates. Anal Biochem. 2008;379:127–9. doi:10.1016/j.ab.2008.04.036. (EPS 912 kb)

## Abbreviations

1α,25(OH)_2_D_3_: 1α,25-dihydroxy vitamin D_3_ – Calcitriol
25(OH)D_3_: 25-hydroxy D_3_
ECM: Extracellular matrix
OA: Osteoarthritis
OPG/RANKL: Osteoprotegerin/receptor activator of nuclear factor kappa-B ligand
RA: Rheumatoid arthritis
VDR: Vitamin D receptor
